# Immunogenicity and Protection against *Mycobacterium caprae* Challenge in Goats Vaccinated with BCG and Revaccinated after One Year

**DOI:** 10.3390/vaccines8040751

**Published:** 2020-12-10

**Authors:** Claudia Arrieta-Villegas, Enric Vidal, Maite Martín, Judit Verdés, Xavier Moll, Yvonne Espada, Mahavir Singh, Bernardo Villarreal-Ramos, Mariano Domingo, Bernat Pérez de Val

**Affiliations:** 1 IRTA, Centre de Recerca en Sanitat Animal (CReSA, IRTA-UAB), Campus UAB, 08193 Bellaterra, Spain; enric.vidal@irta.cat (E.V.); maite.martin@irta.cat (M.M.); Mariano.Domingo@uab.cat (M.D.); bernat.perez@irta.cat (B.P.d.V.); 2 Departament de Medicina i Cirurgia Animals, Universitat Autònoma de Barcelona (UAB), 08193 Bellaterra, Spain; juditverdesmartinez@gmail.com (J.V.); Xavier.Moll@uab.cat (X.M.); Ivonne.Espada@uab.cat (Y.E.); 3 Lionex Diagnostics and Therapeutics GmbH, D-38126 Braunschweig, Germany; info@lionex.de; 4 Animal and Plant Health Agency (APHA), Addlestone KT15 3NB, UK; bev10@aber.ac.uk; 5 Department of Biological, Environmental and Rural Sciences, University of Aberystwyth, Aberystwyth SY23 3DA, UK; 6 Departament de Sanitat i Anatomia Animals, Universitat Autònoma de Barcelona (UAB), 08193 Bellaterra, Spain

**Keywords:** BCG, revaccination, duration of immunity, goat, tuberculosis, vaccine, diagnosis, interferon gamma, antigen-specific memory T-cells

## Abstract

Vaccination has been proposed as a supplementary tool for the control of tuberculosis in livestock. The long-term immunogenicity elicited by bacillus Calmette–Guerin (BCG) and the efficacy of revaccination were investigated in thirty goat kids distributed into three groups: unvaccinated controls, BCG (vaccinated at week 0) and BCG-BCG (vaccinated at weeks 0 and 56). Sixty-four weeks after the first vaccination, all animals were challenged with *Mycobacterium caprae* and examined post-mortem (pathology and bacterial load) at week 73. Antigen-specific interferon-gamma (IFN-γ) release was measured throughout the experiment. At week 59, peripheral blood mononuclear cells were stained for CD4, CD45RO and IFN-γ to determine the presence of antigen-specific cells secreting IFN-γ. The BCG-BCG group showed reductions in rectal temperatures, *M. caprae* DNA load in pulmonary lymph nodes (LN), the volume of lesions in pulmonary LN, mineralization in lungs, and higher weight gains compared to unvaccinated controls. IFN-γ responses were undetectable from 32 weeks after primary vaccination until revaccination, when the BCG-BCG group showed detectable IFN-γ production and a greater percentage of antigen-specific CD4^+^CD45RO^+^IFNγ^+^ and CD4^−^CD45RO^+^IFNγ^+^ cells compared to the BCG and control groups, which may be an indicator of the mechanisms of protection. Thus, re-vaccination of goats with BCG appears to prolong protection against infection with *M. caprae*.

## 1. Introduction

The control of animal tuberculosis (TB) due to *Mycobacterium bovis* and *Mycobacterium caprae*, both members of the *Mycobacterium tuberculosis* complex (MTBC), remains a major challenge not only for animal but also for public health. The WHO estimated that zoonotic TB due to *M. bovis* caused more than 140,000 new human infections and more than 12,000 deaths worldwide in 2018 [1]. In particular, TB-infected goats pose a risk of transmission for people in close contact with animals, such as farmers, veterinarians, or slaughterhouse personnel [2], and have been shown to be a source of infection for other livestock animals [3,4]. In Spain, the EU country with the second highest number of goat heads (2.7 million) and the eighth largest producer of goat milk worldwide (more than 450,000 tons) (data extracted from FAOSTAT on 30/04/2020), caprine TB also causes significant economic losses for animal holdings due to production losses and commercial restrictions imposed on infected herds. The *M. bovis* bacillus Calmette–Guerin (BCG), an attenuated strain of *M. bovis*, is the only licensed vaccine against human TB, and no vaccines against TB are currently registered for livestock. However, over the past two decades, there has been a renewed interest in the use of vaccines to prevent TB in livestock, and several TB vaccination trials have been undertaken in cattle [5]. However, although experimental vaccination trials in cattle have provided encouraging results, cattle field trials have been more variable (reviewed in [5,6]). Our research group and others have undertaken vaccination trials in goats and demonstrated that BCG conferred a degree of protection against *M. caprae* in experimental and natural settings [7,8].

The induction of protective immunity and its duration are key points for vaccine development. Antigen-specific immune responses to mycobacteria are generally measured through the evaluation of the in vitro secretion of Interferon-gamma (IFN-γ) by lymphocytes cultured with MTBC antigens. A requisite for a successful vaccine is the induction and differentiation of T-cells into IFN-γ secreting antigen-specific memory cells that may rapidly reach the site of the infection [9,10]. In addition, the durability of this vaccine-induced immunity is thought to be critical for the development of effective vaccination strategies against TB, enabling the host to respond more quickly and effectively after re-exposure to MTBC antigens. It has been shown that protection conferred by BCG in cattle wanes with time [11]; and that BCG revaccination prolonged the duration of protection conferred against *M. bovis* infection by the primary vaccination [12].

Finally, the major constraint for the use of BCG either in cattle or goats is the interference of vaccination with the tuberculin skin test and IFN-γ release assay (IGRA), which are the approved tests for the diagnosis of TB in livestock. In this sense, the suitability of other MTBC-specific antigens as potential diagnostic test targets for differentiating infected from vaccinated animals (DIVA tests) is another focus of interest on animal TB research [13].

In the present study, we evaluated: (1) the efficacy of BCG revaccination against *M. caprae* challenge in goats; (2) the duration of the immunity induced by BCG vaccination in goats and characterize the immunity induced after revaccination; and (3) the long-term BCG-induced interferences on TB immunodiagnosis.

## 2. Materials and Methods

### 2.1. Animals, Vaccination Schedule and Experimental Infection

Thirty Murciano-granadina female goat kids, aged 6 to 7 weeks, were sourced from the experimental farm of the *Universitat Autònoma de Barcelona* (Catalonia, Spain). Experimental animals were negative to the IGRA as performed using a commercial kit (ID screen^®^ Ruminant IFN-γ, ID.vet, Grabels, France). Goats were randomly assigned to three experimental groups of 10 animals each: Unvaccinated control, BCG, and BCG-BCG. Animals from BCG and BCG-BCG groups were subcutaneously inoculated (right axilla) with 0.5 mL (5 × 10^5^ Colony Forming Units/animal) of *M. bovis* BCG Danish 1331 strain (ATCC, Ref. 35733TM) at week 0. BCG-BCG animals were re-vaccinated 56 weeks later. The BCG vaccine stock was generated as described previously [14] and diluted in sterile phosphate buffered saline (PBS) to reach a suspension of 1 × 10^6^ Colony Forming Units (CFU)/mL before use, and 0.5 mL were inoculated into each animal. Blood samples from all animals were taken every 8 weeks from week 0 to week 56, and at week 59 (3 weeks after re-vaccination).

At week 63, all animals from the three experimental groups (unvaccinated control, BCG, and BCG-BCG) were transferred from the experimental farm to the Biosafety Level 3 (BLS3) facilities of IRTA-CReSA. Animals were housed in groups of 10 with three or four animals from each group. One week later, goats were sedated intramuscularly (with Equipromacina^®^ at 0.05 mg/kg and Torbugesic^®^ at 0.2 mg/kg). Subsequently, goats were anaesthetized intraveonusly (with Propofol Lipuro^®^ at 4–5 mg/kg), and then endobronchially challenged with *M. caprae* (~3 × 10^3^ CFU), as previously described [3]. The inoculum stock of *M. caprae* strain SB0416 (www.Mbovis.org) was prepared as described previously [3]. After the challenge, animals were monitored daily for clinical signs of TB, weighed and bled every two weeks, and rectal temperature was measured every week. Animals were euthanized at week 72 (9 weeks after challenge).

Animals were fed with hay and food supplements as necessary and maintained with water ad libitum throughout the experiment.

### 2.2. Ethics Statement

All experimental animal procedures were approved by the Animal Research Ethics Commission of the *Generalitat de Catalunya* (procedure number 8697/2015), according to current European legislation for the protection of experimental animals (86/609/EEC, 91/628/EEC, 92/65/EEC and 90/425/EEC).

### 2.3. In Vitro IFN-γ Release Assay (IGRA)

Whole Blood samples from all experimental goats were collected from the jugular vein in 10 mL heparinized tubes at weeks 0 (prior to vaccination), 8, 16, 24, 32, 40, 48, 56 (before revaccination), 59, 64 (prior to challenge), 66, 68, 70 and 72 of the experiment. Whole blood samples were stimulated in 96-deep well cell culture plates (Eppendorf Ibérica, Madrid, Spain) with *M. bovis* (PPD-B) and with *M. avium* (PPD-A) tuberculins (CZ Veterinaria, Porriño, Galicia, Spain), and a cocktail of the recombinant proteins ESAT-6 and CFP-10 (EC) [15], at a final concentration for all three reagents of 20 µg/mL. PBS was used as a negative control for the stimulation of blood samples from each animal. Samples were incubated at 37 °C in a 5% CO_2_, 95% humidity atmosphere. After 16–20 h of incubation, plasma supernatants were harvested by centrifugation at 18× *g* for 10 min and analyzed immediately by sandwich ELISA for bovine IFN-γ (ID.vet) or stored at −20 °C for subsequent analysis. The IGRA was performed following the manufacturer instructions. The results of IGRA based on tuberculins were interpreted using the cut-off point of sample to positive ratios (S/P) recommended by the manufacturer: ((Optical density (OD) of PPD-B − OD of PPD-A)/(OD mean kit positive control (CP) − OD mean kit negative control (CN))) × 100. A sample was considered positive if S/P ≥ 16%. The results of IGRA based on EC, were calculated as follows: S/P = ((OD of EC − OD of PBS)/(OD CP − OD CN)) × 100. A sample was considered positive if the sample S/P ≥ 16%.

### 2.4. Flow Cytometry and Intracellular IFN-γ Staining

Flow cytometry analysis was performed at week 59 (3 weeks after revaccination), and in order to evaluate IFN-γ^+^ T-cell memory subsets expanded after revaccination and one year after vaccination. Firstly, the blood from the 30 goats was collected using the BD vacutainer CPT with sodium citrate (BD, Franklin Lakes, NJ, USA). Peripheral blood mononuclear cells (PBMC) were then isolated from blood samples following the BD vacutainer CPT instructions. Isolated PBMC were stimulated in 24-well plates (2 × 10^6^ cells/well) with PPD-B at a final concentration of 10 μg/mL in RPMI 1640 cell culture medium (Sigma-Aldrich, St. Louis, MO, USA) supplemented with 10% foetal calf serum (Sigma-Aldrich), non-essential amino-acids (Sigma-Aldrich), 5 × 10^−5^ M 2-mercaptoethanol, 100 U/mL penicillin and 100 μg/mL streptomycin sulphate. Cells were incubated at 37 °C with 5% CO_2_, 95% humidity for 6 h. Then, brefeldin A (Sigma-Aldrich, Steinheim, Germany) was added at a final concentration of 10 μg/mL, and cells were incubated for a further 16 h.

Stimulated PBMC were counted, and 3 × 10^5^ cells/well were dispensed in 96-well plates. Cells were stained with mouse monoclonal antibodies (mAb) CC8 (IgG2A, conjugated to FITC), which recognizes bovine CD4, and mAb IL-A116 (IgG3, conjugated to RPE), which recognizes bovine CD45RO (both from Bio-Rad Laboratories Inc., Hercules, CA, USA). CD4 and CD45RO stained cells were then fixed and permeabilized with Leucoperm^TM^ (Bio-Rad), according to the manufacturer’s instructions, and incubated with mAb CC302 (IgG1, conjugated to Alexa Fluor^®^ 647, Bio-Rad), which recognizes bovine IFN-γ, for 30 min at room temperature. Finally, cells were re-suspended in 200 µL PBS with 1% paraformaldehyde, stored at 4 °C in the dark, and analyzed within 24 h.

**Figure 1:**
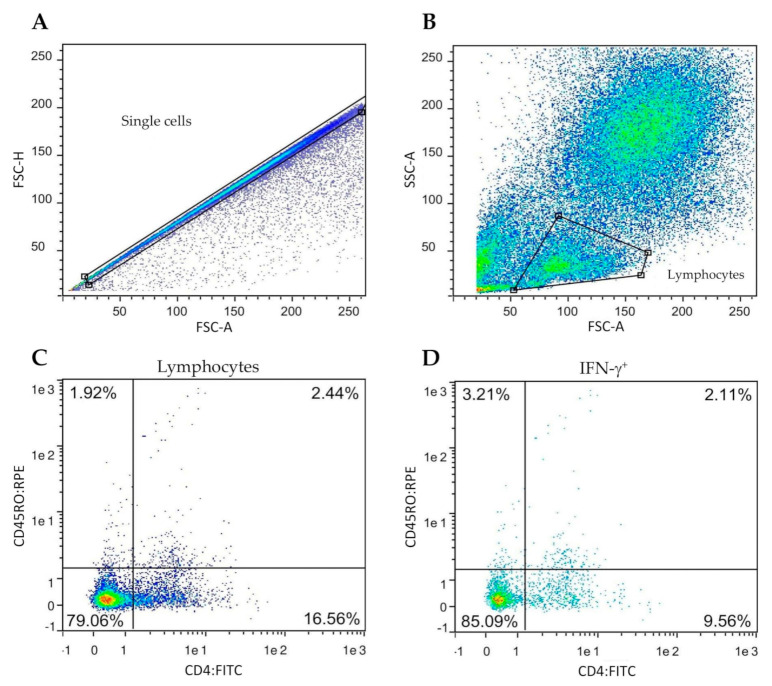
Gating strategy for the determination of frequency of the IFN-γ positive lymphocyte subsets. Peripheral blood mononuclear cells (PBMC) from all goats were cultured in the presence of *M. bovis* tuberculin (PPD-B). (**A**,**B**) Singlet lymphocytes were identified based on the degree of cellular differentiation determined by forward scatter (FSC) and side scatter (SCC). (**C**) Representative frequencies of the CD4/CD45RO cell populations. (**D**) Representative frequencies of intracellular IFN-γ staining of cells gated from prelabelled CD4^+^CD45RO^+^.

Stained cells were analyzed by flow cytometry in a MACSQuantify™ instrument (Miltenyi Biotec, Bergisch Gladbach, Germany). The gating strategy is shown in Figure 1.

### 2.5. Skin Tests

In order to evaluate the effect of vaccination on the skin test, tuberculin-based and DIVA skin tests were carried out in all experimental goats from each group. Skin tests were carried out 59 weeks after vaccination for the BCG group and 3 weeks after revaccination for the BCG-BCG group. First, the proximal zone of both sides of the neck was shaved, and basal skinfold thickness (BST) was measured with a caliper. Then, the single intradermal cervical tuberculin test (SIT) was performed by inoculating 100 µL of PPD-B (CZ Veterinaria, 25,000 IU/mL) at the right side of the neck using a Dermojet^®^ syringe. At the left side of the neck, 100 µL of a tetravalent fusion recombinant protein (TFP, Lionex) was intradermically inoculated to all animals (100 µg/mL). The TFP contained four MTBC-specific antigens (Rv3615c, Rv3020c, ESAT-6, and CFP-10), and was used as DIVA skin test reagent. Finally, 72 h after inoculation, skinfold thickness was measured and compared to BST. An animal was considered as positive if the difference between the BST and the 72 h post-inoculation skinfold thickness was ≥4 mm or clinical signs were seen (i.e., swelling, oedema, exudation, or necrosis), as a doubtful if this difference was ≥2 mm and <4 mm, and negative if the difference was <2 mm.

### 2.6. Post-Mortem Examination

In order to assess the efficacy of BCG revaccination against *M. caprae* challenge, a complete necropsy was carried out at week 72 (week 9 post-challenge). First, goats were euthanized by intravenous injection of an overdose of sodium pentobarbital. Then, pulmonary lymph nodes (LN), i.e., cranial and caudal mediastinal LN and tracheobronchial LN, were carefully removed, avoiding piercing of the pleura. Pulmonary LN were sliced at 3-mm intervals, and diameters of TB lesions were measured for further calculation of the approximate volume of gross lesions. The volume of lesions in the LN was calculated by using the formula of the most similar geometrical morphology of each lesion (sphere, cylinder, cone or prism). Afterwards, whole pulmonary LN were stored at −20 °C for further MTBC DNA burden assessment.

Whole lungs were carefully removed and filled with 10% buffered formalin [16], and analyzed 30 days later by computed tomography following procedures previously described [14]. Briefly, whole lungs were scanned using a 16-slice multi-detector CT scanner (Brivo CT-385, General Electric Healthcare, Madrid, Spain). The volume of lungs and total volume of TB lesions were then calculated employing the volume rendering, using different density patterns (mineralized, solid and cavitary lesions), and multi-planar 2-D and 3-D reconstructions. The volume of mineralized lesions was calculated using 100–300 Hounsfield units.

Other viscera with gross TB-like lesions were collected and fixed in formalin for histopathological confirmation by Hematoxylin/Eosin staining.

### 2.7. M. caprae DNA Burden Assessment by qPCR

Mediastinal and tracheobronchial LN were thawed and sliced with sterile scissors and subsequently homogenized in 10 mL of sterile distilled water using a homogenizer (Masticator^®^, IUL Instruments, Barcelona, Catalonia, Spain). Homogenates of LN for each goat were pooled, and an aliquot of 200 μL was inactivated at 75 °C for 1 h. In parallel, an aliquot of *M. caprae* strain at 10^8^ CFU/mL, the same used for the challenge inoculum preparation, was also inactivated and then serially diluted ten-fold in order to establish titered standards. Afterwards, DNA from homogenates and the standards was extracted using the DNA EXTRACT VK kit (Vacunek, Derio, Spain) and amplified using a commercial MTBC-specific qPCR kit (TBC-VK kit, Vacunek), both steps according to the manufacturer’s procedures. Amplification was performed in a 7500 fast real-time PCR system (Applied Biosystems, Foster City, CA, USA). A standard curve was established using Ct values of standards (ten-fold dilutions of already titered *M. caprae* inoculum strain), and *M. caprae* CFU genomic equivalents were calculated by the extrapolation of Ct value obtained for each DNA sample.

### 2.8. Data Analysis

A randomized block design was used for data analyses conducted after *M. caprae* challenge (blocking was used to remove the eventual box effects). Differences in the volume of TB lesions in the lungs among groups were assessed by two-way ANOVA followed by a one-tailed post-hoc Tukey test. The volume of lesions in LN and volume of mineralized lesions in lungs were Box-Cox transformed to meet a normal distribution of data, and comparisons among groups were also analyzed by two-way ANOVA followed by a post-hoc Tukey test. Comparisons among treatment groups of bacterial loads inferred by qPCR (previously adjusted to consider the blocking factor by obtaining residuals from box effect with a linear regression model), IFN-γ levels obtained by IGRAs prior to challenge, and frequency of cell subsets obtained by flow cytometry were performed by non-parametric Kruskal–Wallis test followed by a post-hoc Wilcoxon rank-sum test. Mean rectal temperatures, mean body weight changes, and IFN-γ levels against PPD-B and EC after challenge were analyzed by a linear mixed-effect model (LMER), with the box factor as a random effect, in order to take into account missing data from two euthanized animals at week 72 of the experiment. Pairwise comparisons among groups were performed with the function “pairs” from the package “emmeans” from R. A *t*-test was used to compare IFN-γ levels of revaccinated animals 8 weeks after the first vaccination and 8 weeks after revaccination. In addition, Spearman and Pearson correlations were used to evaluating the association between different populations of memory or effector T-cells and post-mortem parameters (i.e., bacterial load measured by qPCR, the volume of lesions in LN, and total volume of lesions) and the association of post-mortem parameters with IFN-γ levels at weeks 68, 70 and 72, the body weight changes at weeks 3, 4 and 5 post-challenge, and rectal temperatures at weeks 3, 4 and 5 post-challenge.

The analysis was carried out using RStudio Team (2019). RStudio: Integrated Development for R. (RStudio, Inc., Boston, MA, USA).

## 3. Results

### 3.1. Immunological Responses after Vaccination and Revaccination

**Figure 2:**
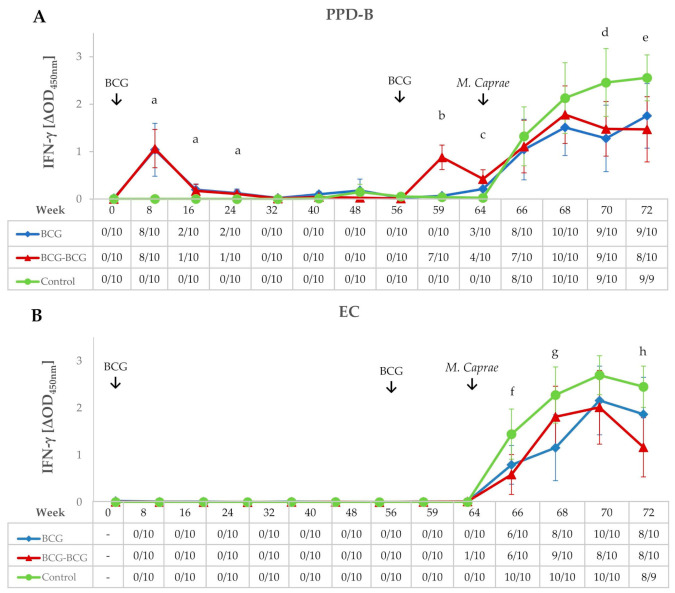
IFN-γ responses after bacillus Calmette–Guerin (BCG) vaccination, revaccination, and *M. caprae* challenge. (**A**,**B**) Mean IFN-γ levels released after stimulation whole blood with *M. bovis* tuberculin (PPD-B) and ESAT-6/CFP-10 (EC) DIVA reagent, respectively. Tables on horizontal axes show IFN-γ release assay (IGRA) qualitative results for each time point. BCG (blue): single BCG-vaccinated group, BCG-BCG (red): BCG revaccinated group, Control (green): unvaccinated group. Kruskal–Wallis test with post-hoc Wilcoxon test: (a) BCG and BCG-BCG compared to Control, *p* < 0.01; (b) BCG-BCG compared to BCG and Control, *p* < 0.001; (c) BCG and BCG-BCG compared to Control, *p* < 0.001; LMER with pairwise comparisons; (d) BCG and BCG-BCG compared to Control, *p* < 0.05; (e) BCG and BCG-BCG compared to Control, *p* < 0.05 and *p* < 0.01, respectively; (f) BCG-BCG compared to Control, *p* < 0.05; (g) BCG compared to Control, *p* < 0.05, (h) BCG-BCG compared to Control, *p* < 0.01.

IFN-γ release after ex vivo stimulation of whole blood with PPD-B and EC antigenic cocktail was measured throughout the experiment (Figure 2A,B, respectively). Both vaccinated groups showed higher secretion of IFN-γ in response to PPD-B at eight weeks post-vaccination (wpv) compared to the control group (*p* < 0.01), and responses decreased appreciably at 16 wpv. At week 32, responses returned to pre-vaccination levels, with all animals remaining negative to IGRA until 56 wpv. Animals in the BCG-BCG group were inoculated at week 56 with BCG as indicated in Materials and Methods. Whole blood IFN-γ responses to PPD-B in the BCG-BCG group increased three weeks after revaccination (59 wpv); these IFN-γ responses were significantly higher compared to non-revaccinated animals (BCG and control groups) (*p* < 0.001). Eight weeks after revaccination (64 wpv), antigen-specific secretion of IFN-γ in the BCG-BCG group was significantly lower than that detected at eight weeks after primary vaccination (mean ΔOD: 0.422, 95% CI: 0.226–0.618; and mean ΔOD: 1.065, 95% CI: 0.660–1.470, respectively, *p* < 0.01, Figure 2A), whereas no animals positive for EC-IGRA were found after vaccination and/or revaccination (Figure 2B).

**Figure 3:**
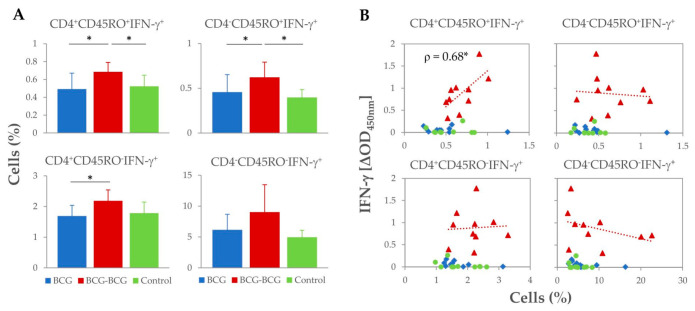
Proportion of *M. bovis* tuberculin (PPD-B)-specific IFN-γ^+^ T-cell subsets isolated from peripheral blood at week 59 (3 weeks after revaccination). (**A**) Frequency (%) of IFN-γ^+^ lymphocyte subsets. * *p* < 0.05, Kruskal–Wallis test with post-hoc Wilcoxon test. (**B**) Correlation of IFN-γ+ lymphocyte subsets frequencies with IFN-γ released after whole blood stimulation with PPD-B at week 59. * *p* < 0.05 (Spearman *ρ* = 0.680).

Analysis of T-cell subsets and potential secretion of IFN-γ was performed by flow cytometry at 59 wpv (three weeks after revaccination). Revaccination of goats resulted in an expansion of all IFN-γ^+^ T-cell subsets after stimulation of PBMC with PPD-B (Figure 3A). Higher frequencies of CD4^+^CD45RO^+^IFN-γ^+^and CD4^−^CD45RO^+^IFN-γ^+^ T-cell subsets (hypothetical IFN-γ-producing memory T-cells) were observed in the BCG-BCG group compared to BCG and control groups (*p* < 0.05), and the proportion of CD4^+^CD45RO^−^ IFN-γ^+^ T-cells was also higher compared to the BCG group (*p* < 0.05). Within revaccinated goats, whole blood PPD-B-specific IFN-γ release at 59 wpv correlated directly with the frequency of CD4^+^CD45RO^+^IFN-γ^+^ populations (Spearman ρ = 0.68, *p* < 0.05), whereas no association between the frequency of the other IFN-γ^+^ cell populations and the levels of IFN-γ released were found (Figure 3B).

**Figure 4:**
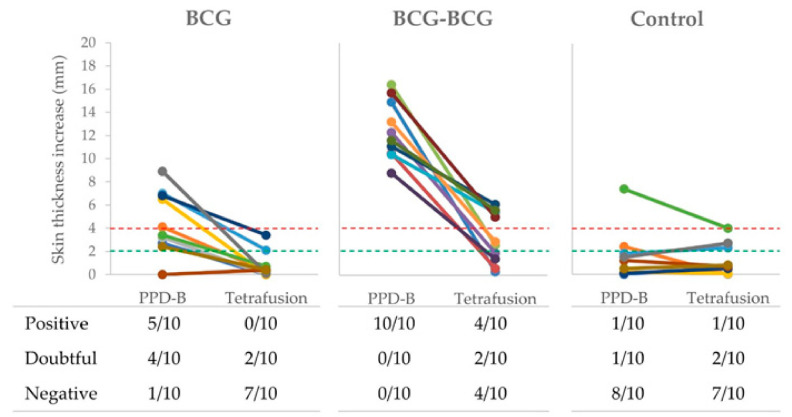
Skin tests. Individual skinfold thickness using *M. bovis* tuberculin (PPD-B) and a Tetrafusion protein (ESAT-6/CFP-10/RV3615c/Rv3020). BCG (single BCG-vaccinated group), skin tested one year after vaccination; BCG-BCG (revaccinated group after one year of first vaccination), skin tested three weeks after revaccination; Control: unvaccinated group. Red dashed line: threshold for positive results in the skin test. Green dashed line: threshold for doubtful results in the skin test. In the table, qualitative results of skin tests are presented per group.

Delayed-type hypersensitivity (DTH), after PPD-B and TFP intradermal inoculations, was also measured in all goats at three weeks post-revaccination (59 wpv). Individual skin thickness increase and qualitative results of SIT and TFP-skin test are shown in Figure 4. Five out of ten and four out of ten single-dose BCG-vaccinated animals were positive and doubtful to SIT, respectively, while 2/10 animals were doubtful to the TFP-skin test. All revaccinated animals were positive to SIT, and 4/10 were positive and 2/10 were doubtful to TFP-skin test. One animal from the control group was positive to both skin tests, and three other animals were doubtful for SIT or TFP-skin tests.

### 3.2. IFN-γ Responses After M. caprae Challenge

Goats were challenged at 64 wpv and remained infected for nine weeks (from 64 to 72 wpv). After the challenge, in vitro whole blood PPD-B-specific IFN-γ responses increased in all groups and reached a plateau in both vaccinated groups from four weeks post-challenge (68 wpv) onwards, while responses remained high in the control group until the end of the experiment, being statistically higher when compared to BCG and BCG-BCG groups (*p* < 0.05) at seven and nine weeks post-challenge (wpc), respectively (Figure 2A). Similar kinetics were observed for EC-specific IFN-γ responses, however a remarkable decrease in the BCG group and even more in the BCG-BCG group were found at 9 wpc. The increase in EC-specific IFN-γ levels (Figure 2B) in the control group was statistically significant (*p <* 0.05) compared to the BCG group at 5 wpc and the BCG-BCG group at 3 and 9 wpc (*p* < 0.05 and *p* < 0.01, respectively).

### 3.3. Clinical Signs and Body Condition Post-Challenge

Goats were monitored for clinical signs of TB after the challenge. Twenty-six, out of 30 animals, showed a sporadic or persistent cough from 4 wpc until the end of the experiment. By the end of the experiment (72 wpv, 9 wpc), severe clinical signs (i.e., dyspnoea, lethargy or anorexia) were observed in 5/10, 5/10, and 5/9 goats in the BCG, BCG-BCG, and control groups, respectively. Dyspnoea and apathy were first observed at 5 wpc in one animal of the control group, which was euthanized one week before the end of the experiment due to ethical endpoint criteria.

**Figure 5:**
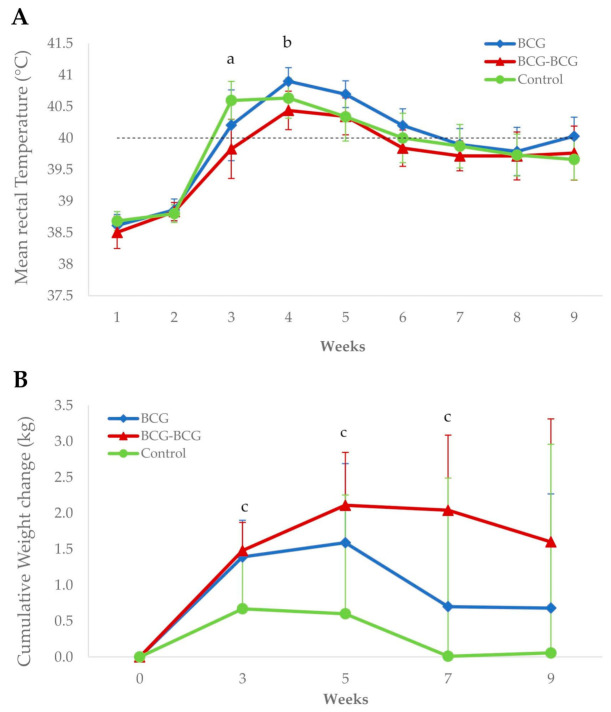
Clinical signs after *M. caprae* challenge. (**A**) Mean rectal temperatures per group. BCG (blue): single BCG-vaccinated group, BCG-BCG (red): BCG revaccinated group, Control (green): Unvaccinated group. Dashed line: fever threshold (40 °C). (a) BCG-BCG compared to Control, *p* < 0.05; (b) BCG-BCG compared to BCG, *p* < 0.05. (**B**) Mean cumulative weight changes per group. (c) BCG-BCG compared to Control, *p* < 0.05; LMER with pairwise comparisons.

Revaccinated animals showed a significantly lower mean rectal temperature (*p* < 0.05) compared to control and BCG groups at 3 and 4 wpc, respectively (Figure 5A). Additionally, the BCG-BCG group showed significant mean body weight gains at 3, 5 and 7 wpc compared to the control group (*p* < 0.05). Mean body weight changes were also higher in the BCG group than in the control group, but were not statistically significant (Figure 5B).

### 3.4. Post-Mortem Findings

**Figure 6:**
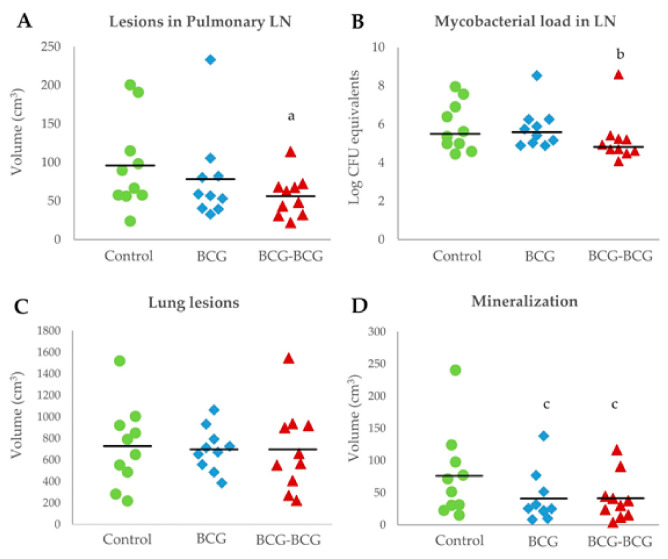
Postmortem analyses. (**A**) Volume of tuberculous lesions in pulmonary lymph nodes (LN). BCG (blue): single BCG-vaccinated group, BCG-BCG (red): BCG revaccinated group, Control (green): unvaccinated group. (a) BCG-BCG compared to Control, *p* < 0.05 (two-way ANOVA with post-hoc Tukey test). (**B**) Mycobacterial load in LN measured by qPCR. (b) BCG-BCG compared to Control, *p* < 0.05, Kruskal–Wallis test with Wilcoxon post-hoc test. (**C**) Volume of TB lesions in lungs. (**D**) Volume of mineralization in lung lesions. (c) BCG and BCG-BCG compared to Control, *p* < 0.1, two-way ANOVA with post-hoc Tukey test.

Animals in the revaccinated group showed a lower mean volume of TB lesions in pulmonary LN, compared to the control (*p* < 0.05, Figure 6A). Bacterial burden, as measured by log CFU genome equivalents, was also lower in the BCG-BCG group compared to unvaccinated controls (*p* < 0.05, after adjustment by box effect, see supplementary data, Figure 6B). However, the mean volume of TB lesions in the lungs was slightly lower in BCG (698 cm^3^, 95% CI: 573–823) and BCG-BCG (698 cm^3^, CI: 453–943) compared to the control group (728 cm^3^, CI: 490–966), but this difference was not statistically significant (Figure 6C). Vaccinated groups also showed a lower mean volume of mineralization in TB lung lesions (BCG: 41 cm^3^, 95%CI: 16–65; BCG-BCG: 41 cm^3^, 95% CI: 19–64) compared to the control group (76 cm^3^, 95%CI: 34–118, *p* < 0.1, Figure 6D). Remarkably, the total volume of lung lesions was not correlated with the volume of mineralization in the lungs (r = −0.019).

The volume of lesions and bacterial DNA load in LN were directly correlated with increased rectal temperature from 3 to 5 wpc (range in which the fever peak was detected) and inversely correlated with body weight increase after challenge (Table 1). Neither post-mortem nor clinical parameters were individually associated with IFN-γ-producing cell frequencies measured before challenge (see Supplementary Materials).

Extra-pulmonary lesions were identified in spleen, liver, kidney, and mesenteric lymph nodes in seven BCG and BCG-BCG goats, and in 9 unvaccinated controls (Table 2).

## 4. Discussion

This study was focused on the effects of BCG revaccination and the duration of immunity afforded by a single-dose vaccination in goats. The results showed that T-cell effector and memory immunity induced by BCG was undetectable one year later, but revaccination with a second dose of BCG one year after primary vaccination, induced cell-mediated responses again at a similar magnitude to that detected at eight weeks after the first immunization and elicited detectable T-cell memory immunity. Moreover, protection after *M. caprae* challenge in re-vaccinated animals was greater than in animals that were not re-vaccinated, as determined by pulmonary lymph node pathology and bacterial DNA load, degree of lung mineralization, fever, and body weight measurements. This is similar to what has been observed in calves vaccinated with BCG at two to four weeks of age, and revaccinated at two years of age, and then challenged with *M. bovis* six months after the second vaccination, whereas single-dose vaccinated animals were not protected [12]. Besides, a field trial in BCG-vaccinated goats has shown an increase in TB incidence one year after vaccination [17], suggesting a loss of protective immunity of BCG after this period of time and the need for a revaccination strategy.

The reduction in bacterial load in pulmonary LN without a reduction in the volume of lung lesions in vaccinated animals is consistent with the findings of a previous study in BCG-vaccinated and *M. caprae*-challenged goats eight weeks later [3]. However, in the present study, the *M. caprae* load in pulmonary LN was approached by measuring DNA equivalents instead of bacterial culture to avoid the negative impact on bacterial counts of viability losses after the congelation and thawing of samples [18]. In addition, quantification by qPCR avoids miscounting due to mycobacterial aggregation and allows the inclusion of known dilutions of the same *M. caprae* strain used for the challenge as a DNA standard, ensuring an accurate equivalent for bacterial counts [19].

Interestingly, vaccinated goats showed a greater reduction in lung mineralized lesions, and this parameter was not directly correlated with the total volume of lung lesions, as observed in a previous study conducted in BCG-vaccinated and *M. caprae*-challenged goats [3]. Hence, despite an extensive lung inflammatory response, BCG vaccination could contain the progress of granulomatous lesions to advanced developmental stages, characterized by necrosis and mineralization [20]. Indeed, the lower proportion of necrotized and mineralized granulomas were previously found in BCG-vaccinated and revaccinated cattle [12]

However, in the present study, 70% of animals in the BCG and BCG-BCG groups showed extra-pulmonary lesions, most of them in the spleen and/or liver, indicating hematogenous dissemination of the infection, whereas in previous experiments, lesions in BCG-vaccinated goats were mainly restricted to respiratory tissues [3,8,18]. In the experiments described above, animals were young and were exposed to *M. caprae* at 0 to 2 months after vaccination; in contrast, in the present study, animals reached adulthood by the time of challenge (approx. 70 weeks old). Thus, the differences in the capacity of BCG to contain the extrapulmonary dissemination of the infection might be explained by sensitization of vaccinated animals with other mycobacteria when the immunity of the primary vaccination waned. Indeed, BCG protection was reduced in cattle naturally pre-sensitized to environmental mycobacteria (EM) [21]. In this regard, it has been suggested that pre-exposure to EM either masks or blocks the effect of BCG vaccination [22]. The fact that four unvaccinated control goats reacted positively to the skin test performed before the challenge, could indicate an exposure of experimental goats to EM during the year that they remained in the experimental farm.

Clinical markers could also be used as indicators of vaccination outcomes. The results in the present study are in concordance with significant reductions in rectal temperatures and body weight increases observed in BCG-vaccinated goats after challenge with *M. caprae* compared to non-vaccinated goats and sheep [3,14].

Understanding of the underlying mechanisms which confer protection is an essential pre-requisite for the development of markers of protection and evaluations of vaccine efficacy. As previously observed in cattle [12], BCG revaccination induced similar levels of PPD-B-specific IFN-γ secreted in peripheral blood compared to those induced by primary vaccination, rather than inducing a classical secondary immune response. Moreover, the significant reduction in IFN-γ after eight weeks of revaccination compared to the IFN-γ levels after eight weeks from the first immunization could be due to the contact with animals with EM. Indeed, in mice orally pre-sensitized with different strains of *Mycobacterium avium*, the IFN-γ responses after BCG vaccination were significantly reduced [23]. On the other hand, a reduction in IFN-γ responses after revaccination could be expected because the goats were adults by the time of revaccination. In fact, it has been demonstrated that specific IFN-γ responses against BCG rapidly decrease with age [24]. However, the strong antigen-specific IFN-γ responses induced by revaccination were not associated with an appropriated protective response, as observed in cattle revaccinated with BCG six weeks after neonatal vaccination [25].

It has been previously reported that the presence of different subsets of memory T-cells elicited by vaccination provided long-term protection in mice and humans [26,27]. In cattle, the presence of CD4^+^CD45RO^+^IFN-γ^+^ and CD8^+^CD45RO^+^IFN-γ^+^ T-cells has been associated with a reduction in the mycobacterial load [28]. Similarly, in the present study, PPD-B-specific IFN-γ-producing memory T-cells, CD4^+^CD45RO^+^IFN-γ^+^ and CD4^−^CD45RO^+^IFN-γ^+^, were highly proliferative in revaccinated goats.

Remarkably, ex vivo antigen-specific production of IFN-γ determined by IGRA was only significantly correlated with the proportion of CD4^+^CD45RO^+^ IFN-γ-producing T-cells in revaccinated goats. Similar findings were observed in BCG-vaccinated and *M. bovis*-infected cattle [29]. The recirculation of antigen-specific IFN-γ-producing memory T-cells has been associated with the containment of mycobacterial replication and/or sterilization of the lesion in the site of the infection [9].

The durability of the antigen-specific memory T-cell response is also required for the development of effective prophylactic strategies against TB. In this study, BCG-induced immunity waned at 59 wpv because no expansion of antigen-specific IFN-γ-producing memory T-cells were observed in goats that received a single dose of BCG. Besides, IFN-γ secreted ex vivo was undetectable from 32 wpv onwards. The persistence of BCG is necessary to induce protective immune responses [30]. In this sense, a previous study in BCG-vaccinated goats demonstrated the complete removal of viable BCG bacilli in the injection site before 24 wpv. It was associated with a decrease in antigen-specific IFN-γ responses [15]. Long-term studies conducted in cattle showed variable whole blood PPD-B-specific IFN-γ detectable responses ranging from less than 50 to over 90 weeks after BCG vaccination [11,12]. Nonetheless, in the latter case, the authors observed a significant decrease in PPD-B-specific IFN-γ-secreting central memory T-cells at 24 months post vaccination (p.v.) compared to 12 months p.v. These results were associated with a decrease in protection against *M. bovis* challenge [11].

To the best of our knowledge, the present study is the first evaluating the duration of the immunity of BCG vaccination in goats. In the light of the results obtained, it is possible that immune protection of BCG in goats waned before one year, as it occurs for other diseases, i.e., clostridial diseases, in which protective immunity decays before then in sheep or cattle, and thus, two or three vaccinations per year are recommended [31,32]. Therefore, revaccination after six months of the first BCG immunization or other schedules needs to be further evaluated in goats. However, it is important to take in account that BCG is not a fully protective vaccine and, as observed with the *Mycobacterium avium* sbsp *paratuberculosis* vaccine, vaccination might be considered as part of the measures for control of the disease in farms [33].

Although single-dose BCG-vaccinated goats showed negative results to IGRA from 32 wpv to the challenge time point, DTH was detected in 50% or 90% of single-dose vaccinated goats at 59 wpv by SIT using the conservative or stringent criteria, respectively. Therefore, BCG did not cause interferences on TB diagnosis by DIVA-based IGRA, as previously demonstrated in goats [3,15], neither in tuberculin-based IGRA seven months after vaccination but compromised the SIT for a minimum of one year. In addition, 20% and 60% of BCG and BCG-BCG-vaccinated goats reacted positively to TFP-ST using the stringent criteria, and cross-reactive responses were also detected in unvaccinated controls. A cocktail including peptides of the four antigens included in TFP was proven highly specific in BCG-vaccinated goat kids [34], but showed divergent sensitivity results in *M. bovis*-infected cattle [35,36]. The lack of specificity found herein does not support the suitability of TFP as a skin test DIVA reagent. In fact, either Rv3615c or Rv3020c are not absent in BCG genome, but the first one cannot be expressed due to the deletion of Esx1 system in BCG [37]. Probably, the presence of Rv3020c in TFP is the causative factor of diagnostic interferences in vaccinated goats. Thus, this antigen could be removed from diagnostic reagents. Additionally, a newly defined skin test containing ESAT-6, CFP-10 and Rv3615c peptide cocktail improved the skin test for the diagnosis of TB in cattle and showed DIVA capability [38].

Finally, during the present study, the main limitation encountered was that the animals, despite being housed in a controlled TB free experimental farm, maintained in the same pen without grazing, and with regular health checking, contact with environmental mycobacteria could not be avoided. Moreover, we cannot rule out the effects of underexposure to sunlight (i.e., deficiency in vitamin D) or other key factors that may have had an effect in mounting an appropriate immune response. Thus, even if the dose for *M. caprae* challenge was similar to that used for previous challenges, the model of adult goats that developed a large volume of lesions in the lungs and vaccinated goats could not properly contain the infection as observed in extrapulmonary lesions. Therefore, future experimental studies in adult goats might better control the aspects mentioned above or use lower doses for the challenge.

## 5. Conclusions

BCG vaccination of goat kids did not provide significant levels of protection against *M. caprae* challenge 64 weeks later, and lifespan for immunity elicited by vaccination was lower than 59 weeks. Moreover, BCG-vaccinated goats showed negative results to the tuberculin-based whole blood IGRA from 32 weeks after vaccination. In contrast, BCG revaccination 56 weeks after primary vaccination was shown to induce an increase in the proportion of antigen-specific IFN-γ-producing memory T-cell phenotypes. BCG re-vaccination was also shown to afford a higher degree of protection against TB in terms of reduced pathology, bacterial DNA load and clinical signs after *M. caprae* infection. Further studies will establish the most suitable moment for BCG revaccination and will determine the durability of revaccination-induced immunity.

## Figures and Tables

**Table 1:** Individual association between clinical and post-mortem parameters.

Post-Mortem Parameter	Body Weight Change				Rectal Temperature		
	W 3	W 5	W 7	W 9	W 3	W 4	W 5
*M. caprae* CFU equivalents	−0.061	−0.221	−0.234	−0.3035	0.234	0.27	0.336
Vol Lesions in LN	−0.414 *	−0.511 **	−0.576 ***	−0.542 **	0.444 *	0.428 *	0.435 *
Vol Lung Lesions	−0.319	−0.482 **	−0.587 ***	−0.692 ***	0.277	0.456 *	0.548 **

Values are Pearson correlation coefficients (r). W: week. CFU: Colony forming units, LN: Pulmonary lymph nodes * *p* < 0.05, ** *p* < 0.01, *** *p* < 0.001.

**Table 2:** Extrapulmonary lesions.

Animals with Extrapulmonary Lesions		Localization of Lesions				
		Spleen	Liver	Ln Ms ^1^	Kidney	Ln RF ^2^
BCG	7/10	5	2	4	2	0
BCG-BCG	7/10	6	2	4	1	4
Control	9/10	8	4	8	4	1

^1^ Mesenteric lymph node. ^2^ Retropharyngeal lymph nodes.

## Supplementary Materials

The raw data used in the present study are available online at https://www.mdpi.com/2076-393X/8/4/751/s1.
